# Vibration Resistance Optimization of Housing Based on CCD and Multi-Objective PSO

**DOI:** 10.3390/mi17020264

**Published:** 2026-02-19

**Authors:** Lei Cheng, Bingxing Wei, Xuanjun Dai, Yanan Bao

**Affiliations:** 1 College of Mechanical and Control Engineering, Guilin University of Technology, Guilin 541000, China; weibingxing@jcgjd.org.cn; 2 Jincheng Research Institute of Opto-Mechatronics Industry, Jincheng 048000, China; baoyanan@jcgjd.org.cn; 3 Shanxi Province Engineering Research Center of Micro/Nano-Structured Materials and Laser Devices, Jincheng 048000, China; 4 Shanxi Key Laboratory of Advanced Semiconductor Optoelectronic Devices and Integrated Systems, Jincheng 048000, China

**Keywords:** semiconductor laser diode array beam combining system, vibration, central composite design method, multi-objective particle swarm optimization algorithm

## Abstract

To improve the operational reliability of semiconductor laser diode array beam combining systems under vibration conditions, this study introduces an integrated optimization approach combining central composite design (CCD) with multi-objective particle swarm optimization (PSO). The methodology involves establishing a response surface model correlating housing stiffener parameters with vibration response indicators, subsequently applying multi-objective PSO for Pareto front optimization. This integrated strategy enables balanced multi-objective optimization of the anti-vibration structure. By modifying the original design into a vibration-resistant configuration, the approach delivers substantial performance enhancements: significantly increased first-order natural frequency, effectively suppressed maximum deformation under random vibration, and well-controlled mass addition. Comparative results demonstrate remarkable improvements over the initial design. The optimized parameter set elevates the first-order natural frequency from 356.3 Hz to 1036.1 Hz while reducing maximum deformation at critical positions from 0.2618 mm to 0.055 mm, with a minimal mass increase of merely 165.47 g. Vibration environment simulation verification demonstrates that after optimization, the output laser power decreases by only 3.3%, and the peak irradiance drops by 5.3%. These improvements substantially enhance system reliability under demanding mechanical conditions, confirming the effectiveness and engineering applicability of the CCD-PSO methodology for anti-vibration design in precision opto-mechanical systems.

## 1. Introduction

Semiconductor lasers have significant advantages over conventional lasers. These advantages include their compact size, high electro-optic conversion efficiency, reliability, and broad wavelength range [1]. Consequently, they are widely used across industrial, medical, military, and communications fields. Currently, substantially increasing the output power of a single semiconductor laser remains challenging. This difficulty is due to inherent limitations, such as thermal damage and nonlinear effects [2]. Laser beam combining systems address this limitation by enhancing the output power of semiconductor lasers. These systems integrate multiple output beams via beam combining technology, which also significantly improves overall application performance [3].

The inherent structural constraints of semiconductor lasers cause significant differences in the emitted light’s characteristics. These include divergence angles along the fast and slow axes, spot size, and beam quality. Consequently, the output laser beam must undergo shaping through optical components before further operations, such as laser beam combining, can be performed [4]. The primary beam shaping process involves two main steps. First, collimating lenses are used to collimate the fast and slow axes of the laser. Second, optical elements homogenize the beam quality of these axes [5]. Both laser beam shaping and combining require precise coordination between the laser diode array and the precision optical components. Furthermore, numerous interfering factors in the service environment can adversely affect beam combining performance. Statistics indicate that approximately 55% of failures in electronic devices under service conditions are vibration-related [6]. The housing acts as the critical external carrier of the laser beam combining system. Its design plays a decisive role in determining whether the system can maintain excellent operational reliability under various complex and demanding mechanical environments. Therefore, investigating the influence of the housing structure on the system’s operational reliability is particularly important.

Research on the mechanical environment reliability of electronic systems has progressively established a multi-level analytical framework. This framework spans from microscopic components to macroscopic systems. Brent experimentally investigated how particle impact damping attenuates the vibration response of equipment assemblies. His work covered the analysis and suppression of vibration transmission paths from individual components to the chassis level [7]. It provided an engineered solution for optimizing dynamic response transfer, specifically for vibration control in aerospace equipment like NASA launch vehicles. Building on this foundation, specialized research by Cui et al. on airborne electronic equipment confirmed a critical finding. Their work demonstrated that the dynamic response and failure mechanisms of printed circuit board assemblies under thermal cycling, random vibration, and combined loads constitute the core factor determining overall device reliability [8]. Notably, Rajaguru et al. conducted dedicated research on power modules, revealing a key insight. They found that the accumulation of vibrational stress—particularly mechanical stress excited at resonant frequencies—can contribute to failure to a much greater extent than thermo-mechanical loads [9]. Simultaneously, Doruk et al. established a systematic simulation-experimental workflow. This workflow is used for designing and verifying housing structures for space optical equipment, with a focus on suppressing vibration-induced deformation through stiffener optimization [10]. Tan et al. developed a random vibration model for tapered flexible cylindrical shells under distributed white noise excitation. This model provides a critical theoretical framework for conducting frequency-domain response analysis of complex continuum structures [11]. For analyzing nonlinear systems under random vibration, Wu et al. introduced the Volterra series. Their work builds upon existing frequency-domain theory to systematically develop a power spectral density (PSD) analysis framework tailored for nonlinear systems [12]. Sheng et al. studied the frequency-domain analysis and dynamic reliability assessment of non-classically damped linear structures under non-Gaussian random excitation. Their study established a more general theoretical framework [13]. This work significantly expanded the applicability boundaries of traditional random vibration theory. It now addresses scenarios involving complex coupled conditions of damping characteristics and excitation types. By developing higher-order frequency-domain methods that go beyond classical PSD analysis, this collective research provides vital theoretical tools and methodological support. These tools address the challenges of accurately predicting vibration response and assessing dynamic reliability for opto-mechanical systems. This includes systems with significant non-proportional damping or those subjected to non-Gaussian excitations. However, a significant research gap remains. Despite the valuable insights from existing research on structural protection, there is still a lack of in-depth and systematic investigation into the failure modes and reliability enhancement strategies for specialized systems. Semiconductor laser diode array beam combiners are a prime example, as they feature intricate internal optical structures and are particularly sensitive to vibration.

This study initiated the research by establishing a three-dimensional model of the semiconductor laser diode array beam combining system. Finite element analysis software was employed to perform modal and random vibration analyses on the beam combining module, determining the system’s modal frequencies and characterizing its response under vibrational loads. Subsequently, a CCD methodology was adopted, utilizing the width and height of the stiffeners on the housing base as design variables. The first-order natural frequency, mass, and deformation under random vibration were defined as the evaluation metrics, systematically elucidating the influence laws of the structural parameters on the system’s performance and successfully constructing a high-precision response surface model. Building upon this foundation, the Multi-Objective PSO algorithm was innovatively integrated with the CCD framework to perform optimization search on the established model. This integrated approach yielded the optimal parameter combination for the housing stiffeners, thereby achieving a multi-objective collaborative optimization that effectively balances vibration resistance, weight reduction, and deformation control. The proposed method demonstrated a significant enhancement in the stability of the beam combining system under vibration conditions. Its effectiveness has been validated through vibration environment simulation verification. This work provides a reliable and portable solution for the anti-vibration design of similar precision opto-mechanical structures, offering substantial practical value for engineering applications.

## 2. Numerical Models and Theoretical Analysis

### 2.1. Physical Geometric Model

**Figure 1:**
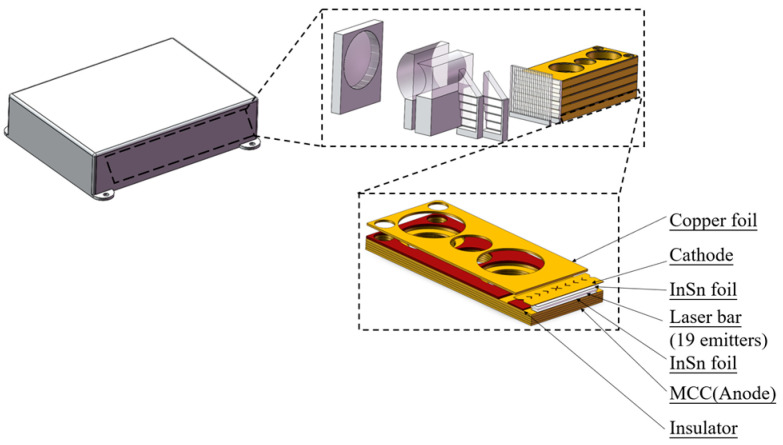
Semiconductor laser diode array beam combining module model.

A solid model of the semiconductor laser diode array beam combining module was created using 3D modeling software (SolidWorks 2022), with dimensions of 100 mm × 140 mm × 33 mm, as shown in Figure 1. The housing material is 6061-T6 aluminum alloy(Washington, DC, USA). Key physical properties of this material, including density, Young’s modulus, and Poisson’s ratio, are listed in Table 1. The finite element model discretizes the housing structure by using a combination of triangular and quadrilateral shell elements. Tetrahedral elements are employed to fill more complex three-dimensional regions. Mesh generation is driven by an intelligent algorithm. Mesh quality is ensured by controlling element density, edge length, and conformity to geometric curvature.

This study focuses solely on how the housing structure influences the operational reliability of the semiconductor laser diode array beam combining module under vibration conditions. The housing itself contains various internal devices and components. Modeling these details would complicate the subsequent finite element analysis. Therefore, the following assumptions are made to simplify the computational analysis:

(1) The mechanical properties within the module housing, such as density, stress, displacement, and strain, are continuous.
(2) The module housing is composed of a uniform material, with identical physical properties throughout its structure.
(3) Components with local features that are non-critical to the optical path, such as bolts and screw holes, are simplified in the model.

### 2.2. Mesh Independence Verification

To ensure the reliability and stability of the finite element analysis results, a mesh independence verification was performed. This verification aimed to optimize computational costs and balance precision with efficiency. It was conducted on the finite element model of the semiconductor laser diode array beam combining module under identical conditions. Table 2 presents the corresponding numerical solutions. These include the first-order natural frequency and the maximum deformation at the housing base under different mesh counts. When the mesh count reaches 101,490, the maximum error is only 0.3527%. This error rate remains below 0.4%, thereby satisfying the requirement for mesh independence. The final discretization scheme was determined following a comprehensive assessment. This assessment considered mesh quality, numerical solution error, and computational cost. Based on this, a mesh count of 101,490 was deemed optimal for the semiconductor laser diode array beam combining module.

### 2.3. Theories of Vibration

#### 2.3.1. Theory of Modal Analysis

Modal analysis employs vibration theory to investigate the fundamental vibration characteristics of mechanical structures. The semiconductor laser diode array beam combining module constitutes a complex vibrational system. This system is typically abstracted as a multi-degree-of-freedom system. Accurately establishing the system’s differential equations of motion provides the theoretical cornerstone. This foundation is essential for analyzing the dynamic characteristics of the mechanical structure.

The influence of damping can be neglected during modal analysis, with the formula given as [11]:

(1) $$\left[M\right]\left\{\ddot{x}\left(t\right)\right\}+\left[K\right]\left\{x\left(t\right)\right\}=\left\{0\right\}$$

where $\left[M\right]$ is the mass matrix describing the system’s inertial properties; $\left[K\right]$ is the stiffness matrix representing the system’s elastic restoring force.

When the housing structure of the semiconductor laser diode array beam combining module undergoes simple harmonic vibration, its harmonic motion is transformed into

(2) $$\left\{x\left(t\right)\right\}=\left\{X\right\}\sin p\;t$$

where $\left\{X\right\}$ represents the displacement amplitude of each degree of freedom when the system undergoes simple harmonic vibration; p denotes the system’s natural vibration frequency.

Substituting Equation (2) into Equation (1) and rearranging yields the mode shape equation of the system:

(3) $$\left(\left[K\right]-p^{2}\left[M\right]\right)\left\{X\right\}=\left\{0\right\}$$

Equation (3) constitutes a homogeneous system of linear equations. Therefore, the necessary and sufficient condition for it to possess a non-trivial solution is given by Equation (4):

(4) $$\left|\left[K\right]-p^{2}\left[M\right]\right|=0$$

The system characteristic equation (also known as the frequency equation) described by Equation (4) is an n-th order algebraic equation in terms of $p^{2}$. By solving this characteristic equation, the characteristic roots of the system can be obtained. These characteristic roots determine the system’s natural frequencies and its dynamic response characteristics.

By substituting each $p_{i}^{2}$ into Equation (3), the corresponding amplitude column vector ${\left\{X\right\}}_{i}$ can be solved, which represents the system’s principal mode shapes.

#### 2.3.2. Theory of Random Vibration Analysis

Random vibration analysis is fundamentally a frequency-domain method based on linear assumptions. In practical engineering structures, damping plays a critical role in dissipating vibrational energy and suppressing resonance peaks. To render the problem mathematically tractable, the damping matrix $\left[C\right]$ is commonly modeled using a proportional damping approach, such as Rayleigh damping, where it is expressed as a linear combination of the mass matrix $\left[M\right]$ and the stiffness matrix $\left[K\right]$, i.e., $\left[C\right]$ = α$\left[M\right]$ + β$\left[K\right]$. Here, α and β are the proportionality coefficients. In this study, a constant damping ratio of 0.01 with engineering representativeness was selected, following the recommended range provided in the NASA Structural Design Handbook (NASA-STD-5002A). The first two natural frequencies within the analysis bandwidth, which contribute most significantly to the vibrational response, were chosen as control points under the assumption of an identical damping ratio. Subsequently, the specific values of α and β were determined as 30.10 s^−1^ and 2.93 × 10^−6^ s, respectively, by solving the Rayleigh damping equations. Under this formulation, the equation of motion for the system subjected to random excitation is given by [14]:

(5) $$\left[M\right]\left\{\ddot{x}\left(t\right)\right\}+\left[C\right]\left\{\dot{x}\left(t\right)\right\}+\left[K\right]\left\{x\left(t\right)\right\}=\left\{f\left(t\right)\right\}$$

where $\left\{f\left(t\right)\right\}$ represents the external excitation vector. Solving the forced vibration equation, Equation (5) yields the system’s impulse response. When the system’s degrees of freedom are coupled, it is necessary to decouple Equation (5) before solving.

However, for precision opto-mechanical structures exhibiting contact nonlinearities or large deformation effects, linear frequency-domain analysis may be insufficient to accurately predict their dynamic response. These nonlinearities can induce complex dynamic behaviors, such as harmonic responses and jump phenomena. In such cases, a more accurate assessment necessitates recourse to nonlinear vibration theory or advanced numerical methods [12]. While the present study is primarily based on a linear framework to ensure the engineering practicality of the optimization method, the established CCD-PSO framework holds potential for extension to the analysis of nonlinear problems.

Based on the orthogonality of the normal modes with respect to the matrices $\left[M\right]$ and $\left[K\right]$, the coupled system of equations can be decoupled into a series of independent equations by left-multiplying both sides of Equation (5) by the transpose of the normal mode matrix. Selecting a set of generalized coordinates $\left\{q\left(t\right)\right\}$, let

(6) $$\left\{x\left(t\right)\right\}=\left[\tilde{P}\right]\left\{q\left(t\right)\right\}$$

Substituting Equation (6) into Equation (5) and left-multiplying both sides of the equation by ${\left[\tilde{P}\right]}^{T}$ yields the decoupled forced vibration equation:

(7) $$\left[I\right]\left\{\ddot{q}\left(t\right)\right\}+\left[\tilde{C}\right]\left\{\dot{q}\left(t\right)\right\}+\left[\Lambda \right]\left\{q\left(t\right)\right\}=\left\{\tilde{f}\left(t\right)\right\}$$

where $\left[I\right]$ is the identity matrix; $\left[\Lambda \right]$ is the eigenvalue matrix, whose diagonal elements are the squares of the respective natural frequencies; $\left[\tilde{C}\right]$ is the diagonal damping matrix obtained after the modal transformation; and $\left\{\tilde{f}\left(t\right)\right\}$ is the generalized force vector in modal coordinates, obtained by transforming the excitation vector $\left\{f\left(t\right)\right\}$ in physical coordinates using the modal matrix $\left[P\right]$.

Equation (8) represents a nonhomogeneous system of linear equations, in which the *i*-th equation can be written as:

(8) $$\ddot{q}\left(t\right)+c_{i}\dot{q}\left(t\right)+p_{i}^{2}q\left(t\right)=\left\{f\left(t\right)\right\}$$

where $q_{i}\left(t\right)$ represents the i-th principal coordinate. Once the modal coordinate responses for the i-th mode of the system are obtained, the dynamic responses of the system at each physical degree of freedom can be determined through the inverse transformation from normal coordinates.

## 3. Modal Analysis

**Figure 2:**
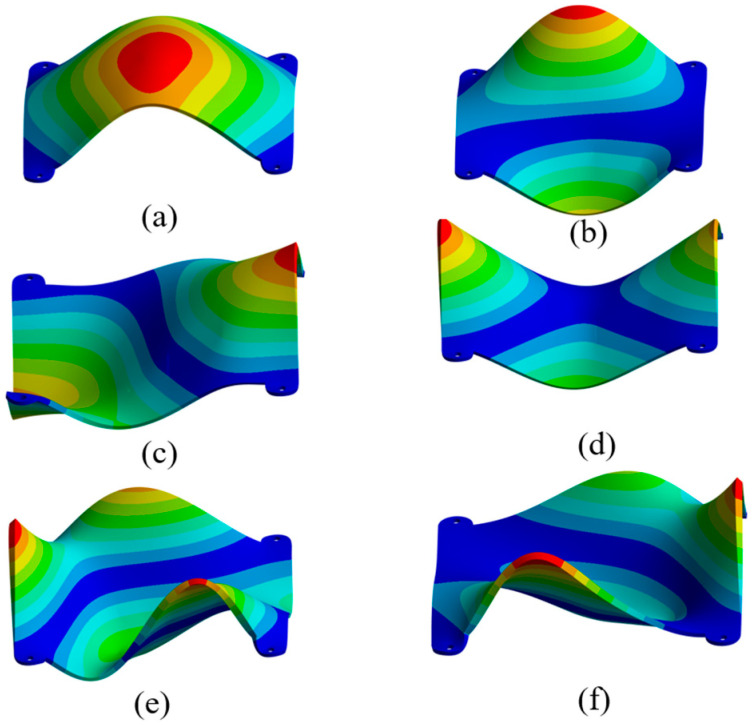
Mode shapes of the semiconductor laser diode array beam combining housing base. (**a**) First-order natural frequency mode shape; (**b**) Second-order natural frequency mode shape; (**c**) Third-order natural frequency mode shape; (**d**) Fourth-order natural frequency mode shape; (**e**) Fifth-order natural frequency mode shape; (**f**) Sixth-order natural frequency mode shape.

Based on vibration theory, modal analysis was performed on the semiconductor laser diode array beam combining module. This analysis used the Modal module within finite element analysis software. The Subspace method was applied for the analysis, and the nodes at screw hole locations were fully constrained. The base of the beam combining module housing supports the laser diode array and various optical components. Vibration-induced deformation directly causes relative displacement between these lasers and optical elements. This displacement leads to the failure of the precisely aligned laser beam combining system. Consequently, it impairs the module’s reliability in service environments. Therefore, particular attention is focused on the deformation at the base of the laser diode array beam combining module housing under random vibration. The first six modal frequencies are listed in Table 3. The corresponding first- to sixth-order mode shapes at the module base are shown in Figure 2a to Figure 2f, respectively.

The structural design of the beam combining module housing must ensure extremely high positional stability and operational reliability. This requirement applies to the internal laser diode array and optical components under long-term service conditions. Therefore, the housing must possess excellent stiffness characteristics. Figure 2a–f present the modal analysis results for the base of the beam combining module housing. These results reveal a critical correlation between the housing’s dynamic characteristics and optical performance. Low-order modal frequencies directly determine the system’s sensitivity to external vibrational excitation. Meanwhile, mode shape characteristics reflect the dynamic displacement distribution at the mounting locations of optical components. This displacement distribution directly impacts the collimation accuracy of the laser transmission path. The vibration patterns at the housing base primarily manifest as multiple depressions or protrusions. The third-order natural frequency is 847.45 Hz, while the second-order natural frequency is 731.05 Hz. The separation between them is only 116.3 Hz. This dense modal distribution makes the system prone to multi-mode resonance. The first-order natural frequency is only 356.3 Hz, which is below the 400 Hz safety threshold. Modal analysis results show that the normalized mode shape displacement indicates a maximum relative deformation of 147.22 mm at the housing base (this value represents the relative distribution of the mode shape, not the actual physical displacement). This mode shape demonstrates that the housing base is prone to significant overall bending deformation at low-order frequencies. More importantly, as shown in Section 4 random vibration analysis, under standard PSD excitation, the maximum physical displacement induced by vibration at critical locations of the housing can reach 0.2618 mm, which far exceeds the focal length tolerance of the fast-axis collimating lens (0.16 mm). This indicates that under vibrational environments, structural deformation transmitted through paths directly disrupts the optical path collimation state. Therefore, structural optimization is imperative to significantly increase the first-order natural frequency and suppress vibration transmission.

## 4. Random Vibration

Random vibration analysis methodologically falls within the scope of spectral analysis [15]. Spectral analysis is a statistical estimation technique. It predicts structural dynamic responses based on modal analysis results and a known excitation spectrum. The essence of this method lies in combining the system’s inherent frequency-domain properties with the statistical energy distribution of external random loads. This combination efficiently predicts structural response characteristics under uncertain loads [16]. The analysis was conducted in accordance with the relevant equipment environmental test method vibration test standard (GJB 150.16A-2009) [17]. Specifically, the PSD analysis function within the ANSYS(ANSYS2023 R1) spectral analysis module was utilized. The acceleration power spectrum, as specified in Table 4, was input for this analysis.

**Figure 3:**
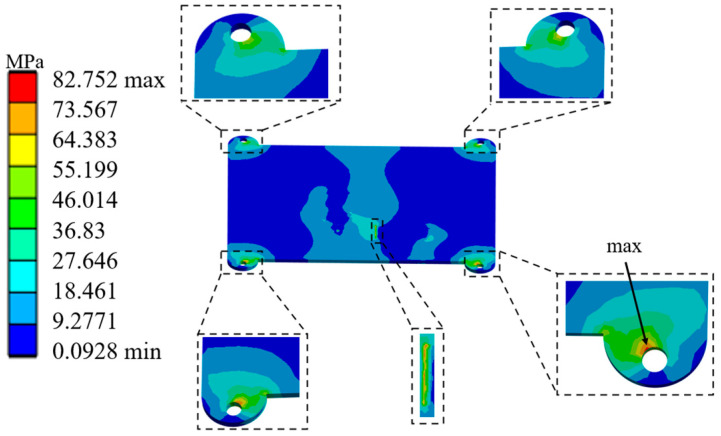
Stress distribution contour of the laser housing base under random vibration.

Figure 3 shows the stress distribution contour plot (3σ) at the base of the laser beam combining module housing under random vibration loads. The analysis indicates that the housing stress exhibits significant local concentration characteristics. The maximum stress value is 82.752 MPa. This maximum stress occurs at the corner regions of the mounting holes for the laser diode array and the optical components. This stress concentration phenomenon is primarily induced by the bending deformation of the base housing structure under random vibration excitation. This finding aligns with the typical mechanical response where stress concentrates in regions of abrupt stiffness change under vibrational environments. Crucially, the vibration-induced structural deformation is directly transmitted to the optical components and the semiconductor laser diode array mounted on it. This transmission causes positional shifts in these elements. The laser beam combining optical path is extremely sensitive to alignment accuracy. Even minute positional deviations are sufficient to disrupt its precisely calibrated state. Such disruptions can lead to issues including beam steering, mode instability, or a significant degradation in combining efficiency. These issues ultimately result in system functional failure. The housing material is 6061-T6 aluminum alloy. With a safety factor of 1.5 applied, its allowable stress is approximately 207 MPa. This safety factor primarily addresses the prevention of material yielding or fatigue failure. Its value is higher than the current maximum stress. This indicates that static strength requirements are satisfied. However, this safety factor does not fully encompass the more stringent requirements. These stricter requirements are imposed by vibration-induced deformation on the positional accuracy of the optical components.

**Figure 4:**
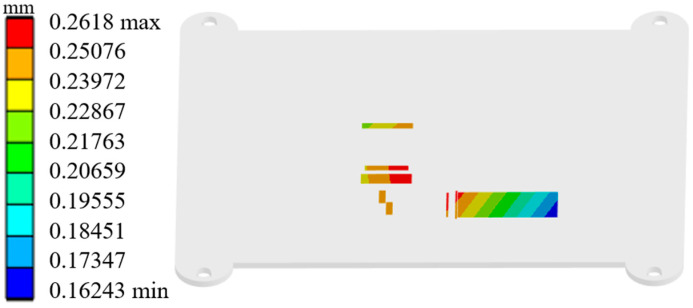
Z-direction deformation contour at critical locations on the base of the laser diode array beam combining module.

The random vibration simulation analysis reveals a significant spatial consistency between the deformation distribution and the stress contour at the base of the laser beam combining module housing. This consistency in mechanical response originates from the vibration energy transfer path and stiffness distribution characteristics within the structure. The deformation response is particularly critical in regions contacting key optical components. This response directly determines the optical path collimation accuracy and beam combining efficiency. Figure 4 shows the 3σ deformation contour at the installation positions of the laser diode array and main optical components within the module under random vibration. During the random vibration test, the maximum deformation at a critical location on the base reached 0.2618 mm. The focal length tolerance of the fast-axis collimating lens is only 0.16 mm. Therefore, the positional accuracy of the fast-axis collimating lens has the most significant impact on the module’s reliability. The housing deformation induced by random vibration caused a displacement of 0.2571 mm in the fast-axis collimating lens. The relative displacement between the fast-axis collimating lens and the laser diode array was imported into optical software after the vibration test. This analysis showed that the combined power dropped from 300 W before the test to 1.5 W. This decrease represents a power reduction of over 10%. The result indicates that the random vibration caused the beam combining module to fail. Therefore, the vibrational environment severely impacts the laser coupling efficiency of the laser diode array beam combining module.

## 5. Structural Optimization

Through modal and random vibration analysis of the laser diode array module housing, it becomes evident that the base of the laser housing directly impacts the reliability of the beam combining module. The existing housing structure cannot ensure the long-term stable operation of the laser diode array beam combining module in service environments. Currently, most studies aim to improve the housing’s vibration resistance by increasing the housing thickness or installing stiffeners. However, increasing the housing thickness significantly adds to the mass of the laser diode array beam combining module. This weight increase compromises the module’s applicability. Furthermore, such approaches often rely on a single evaluation metric. This reliance neglects the influence of the interaction between stiffener structural parameters on the housing’s vibration resistance. It also overlooks the potential misleading nature of relying on a single metric. Additionally, experimental efficiency issues are often ignored. Consequently, these methods fail to effectively enhance the reliability of the laser diode array beam combining module in vibrational environments. The simulation-based approach, substituting for physical experiments, demonstrates advantages in the early design stage, including cost-effectiveness, high efficiency, and ease of parametric studies, effectively avoiding the limitations of traditional trial-and-error methods and providing an efficient pathway for structural optimization.

To systematically enhance the vibration resistance of the semiconductor laser diode array beam combining module, this paper adopts a strategy combining CCD and multi-objective PSO algorithm. This involves a multi-objective optimization design of the key structural parameters of the stiffeners on the base of the beam combining module housing, aiming to achieve a significant improvement in vibration resistance. Compared to traditional optimization algorithms, the combined CCD-PSO strategy demonstrates unique advantages in solving such complex engineering optimization problems. Gradient-based methods, such as gradient descent, heavily rely on the convexity and differentiability of the objective function, struggle to handle highly nonlinear structural dynamic response problems, and are prone to becoming trapped in local optima [18]. While evolutionary algorithms like genetic algorithms possess global search capabilities, they typically require thousands of fitness function evaluations, resulting in prohibitive computational costs [19]. The innovation of the combined CCD-PSO strategy lies in integrating efficient experimental design with intelligent search algorithms. CCD is used to construct a high-precision surrogate model with very few sample points, thereby avoiding the “curse of dimensionality.” Subsequently, the PSO algorithm can perform fast and efficient global Pareto front searches on this lightweight surrogate model. This approach reduces the computational burden of the optimization process by several orders of magnitude while ensuring solution quality.

CCD establishes a mathematical model between design variables and objective functions. This is achieved by fitting experimental data through mathematical functions. This approach captures high-dimensional information with a low number of experimental runs. It effectively handles the linear effects of factors. It also precisely captures nonlinear effects and interaction terms. Furthermore, it balances accuracy and result reliability. However, CCD requires specific point configurations. Factorial points are needed to explore main effects and two-way interactions. Axial points capture nonlinear trends. Center points evaluate experimental error [20]. In contrast, the multi-objective PSO algorithm adopts a parallel search strategy. This strategy simultaneously handles multiple conflicting objectives. The algorithm efficiently explores the global optimum region in high-dimensional solution spaces. It also accurately approximates the distribution of the Pareto front. Nevertheless, the algorithm requires appropriate parameter settings. These include the inertia weight, acceleration coefficients, and crowding distance calculation mechanism. Proper settings balance convergence speed and solution set diversity.

Based on the characteristics of the methods described above, the multi-objective optimization process combining CCD and PSO primarily includes several steps. First, the design variables, constraints, and objective functions of the optimization problem must be clearly defined. Second, response surface analysis is performed on the design variables using CCD. Subsequently, a regression model is constructed based on the data from the CCD. Then, the significance of this regression model is tested through analysis of variance (ANOVA). Finally, the optimal solution on the Pareto front is obtained using the PSO algorithm. This determines the optimal parameter combination under the given constraints [21].

### 5.1. Design Variables and Objective Functions

**Figure 5:**
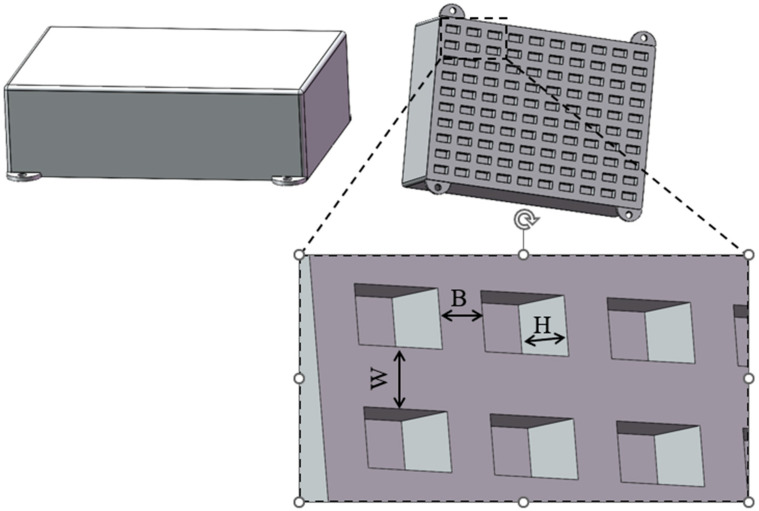
Schematic diagram of the stiffener at the base of the beam combining module housing.

The primary objective of this multi-objective optimization is to enhance the laser diode array beam combining module’s performance. Specifically, it aims to increase the first-order natural frequency and reduce deformation under vibrational environments. The optimization also ensures that any increase in housing mass remains within an acceptable range. These improvements will enhance the module’s reliability during service conditions. Research indicates that adding stiffeners to the housing effectively improves vibration resistance. The main structural parameters of these stiffeners are width and thickness. Figure 5 provides a schematic diagram of the stiffeners on the base of the beam combining module housing. In this diagram, *W* and *B* represent the widths in the horizontal and vertical directions, while *H* denotes the stiffener thickness [22]. Therefore, this study selects width *W*, width *B*, and thickness *H* of the stiffeners as design variables. The objective functions are defined as three key performance metrics. These include the first-order natural frequency of the module, the maximum deformation at the housing base, and the mass of the housing base. The coded level values and actual values for these stiffener parameters are listed in Table 5.

### 5.2. Response Surface Regression Model

Response Surface Methodology (RSM) is a statistical technique for modeling and optimizing processes. It quantifies nonlinear relationships between multiple factors and response values. This quantification requires only a small number of experiments, achieved through rational experimental design and mathematical modeling. RSM efficiently analyzes interactions between factors and optimizes for the best conditions. Its core components include experimental design, model fitting, and ANOVA [23]. Table 6 presents the design scheme from the CCD method. The table also lists the corresponding finite element simulation results. These results are for the beam combining module’s first-order natural frequency, maximum base deformation, and housing base mass.

Based on the aforementioned CCD scheme and results, polynomial approximation fitting is employed to formulate response surface model expressions. These expressions approximate the relationship between the design variables (*W*, *B*, and *H*) and three key objective functions. The objective functions are the first-order natural frequency of the beam combining module (*Y*_1_), the maximum deformation at the key location of the housing base (*Y*_2_), and the mass of the housing base (*Y*_3_). The resulting models are shown in Equations (9)–(11).

(9) $$\begin{array}{c} Y_{1}=171.07088+32.85514W+13.07043B+152.54495H+0.264688WB-0.980625WH\\ -0.428125BH-2.26414W^{2}-0.815160B^{2}-0.699417H^{2} \end{array}$$

(10) $$\begin{array}{c} Y_{2}=0.272756-0.008744W-0.004766B-0.071252H+0.000037WB+0.000956WH\\ +0.000540BH+0.000382W^{2}+0.000167B^{2}+0.006224H^{2} \end{array}$$

(11) $$\begin{array}{c} Y_{3}=54.35470+5.03176W+5.02688B+8.38523H-1.00563WB+2.58875WH\\ +1.37125BH \end{array}$$

**Figure 6:**
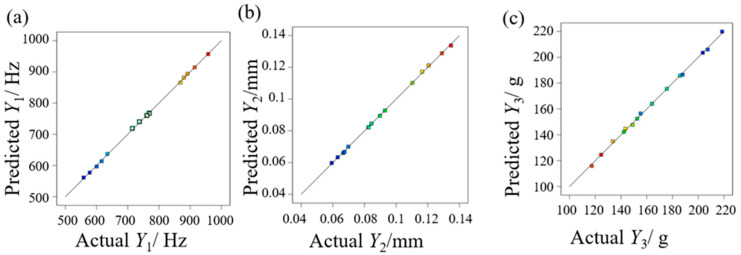
Comparison of actual and predicted values in the response surface regression model. (**a**) First-order natural frequency of the beam combining module; (**b**) maximum deformation at the housing base; (**c**) mass of the housing base.

Figure 6 compares the predicted and actual values for three key metrics: the first-order natural frequency, the maximum deformation at the housing base, and the mass of the beam combining module. The data points are distributed close to the diagonal line. This distribution indicates a strong agreement between the predicted and actual values at the design points. The maximum error between the actual and predicted values is 0.596% for the first-order natural frequency. For the maximum deformation at the housing base, the error is 1.039%. The mass prediction shows an error of 1.142%. These error margins are small. Together, these results demonstrate that the model fits the actual scenario well.

### 5.3. Response Surface Analysis

Model validation is a critical step in the multi-objective optimization integrating RSM and the PSO algorithm. This step directly determines the accuracy of subsequent optimization results. It ensures the reliability of the response surface models. Therefore, an ANOVA is required for the corresponding response surface models. These models predict the first-order natural frequency, the maximum deformation at the housing base, and the mass of the beam combining module.

The ANOVA results for the regression equation of the beam combining module’s first-order natural frequency are presented in Table 7. These results indicate a highly significant model, with a *p*-value less than 0.0001. This significance confirms that the regression model demonstrates a good fit and is valid. Within this model, several terms are identified as significant factors influencing the first-order natural frequency response. These significant terms include *W*, *B*, *H*, *W^2^*, and *B^2^*. Furthermore, the ANOVA results for the regression equation of the maximum deformation at the housing base are shown in Table 8. The results for the housing base mass regression equation are presented in Table 9. These results confirm the validity of both corresponding response surface models. For the maximum deformation model, the significant terms are *W*, *B*, *H*, *WH*, *BH*, *W^2^*, *B^2^*, and *H^2^.* For the housing base mass model, the significant terms are *W*, *B*, *H*, *WB*, *WH*, and *BH*.

Table 10 presents the coefficient of determination (*R^2^*) for the credibility analysis of the response values. Values closer to 1 indicate a more significant correlation for the response surface model [24]. The *R^2^* values are 99.96% for the first-order natural frequency of the beam combining module. For the maximum deformation at the housing base, the *R^2^* value is also 99.96%. The *R^2^* value for the mass of the housing base is 99.89%. The differences between the predicted *R^2^* and the Adjusted *R^2^* are 0.0028 for the first response. For the second response, the difference is 0.0031. For the third response, the difference is 0.0090. All these differences are significantly less than 0.2. These results demonstrate that the response surface models have a significant correlation. The models can accurately reflect the relationship between the response values and the independent variables. Therefore, they serve as effective mathematical tools for predicting three key parameters: the first-order natural frequency, the maximum deformation at the housing base, and the mass of the beam combining module.

### 5.4. Particle Swarm Algorithm Optimization

Based on the constructed response surface models *Y_1_*, *Y_2_*, and *Y_3_*, the PSO algorithm was applied. This optimization targeted three objectives: the first-order natural frequency, the maximum deformation at the housing base, and the mass of the beam combining module. The PSO algorithm is a swarm intelligence optimization technique. It simulates the social behavior observed in bird flocks or fish schools. The algorithm searches for optimal solutions through collaboration and information sharing among individuals within a population. Each particle dynamically adjusts its velocity and direction in the solution space. This adjustment is based on the particle’s own historical best experience and the population’s historical best experience. This mechanism balances global exploration and local exploitation capabilities. Consequently, it achieves efficient solutions for complex optimization problems [25].

The workflow of the PSO algorithm proceeds as follows. First, the particle swarm is initialized. This involves randomly setting the position and velocity of each particle. Initial values are also assigned for the personal best and the global best positions. Then, the fitness value of each particle is calculated. Based on these values, the personal best position and the global best position are updated. Subsequently, the velocity and position of each particle are updated. This update follows the velocity update formula. Finally, the iteration count and loop conditions are checked. If the termination criteria are not met, the above steps are repeated. This process continues until the criteria are satisfied. At that point, the global optimal solution is output [26].

The multi-objective optimization mathematical model is formulated as follows:

(12) $$\left\{\begin{array}{c} Maximize:Y_{1}\left(W,B,H\right)\\ Minimize:Y_{2}\left(W,B,H\right)\\ Minimize:Y_{3}\left(W,B,H\right)\\ Where:Y_{1}\geq 356.3\;Hz\\ Y_{2}\leq 0.2618\;mm\\ Y_{3}\leq 220\;g\\ 1\;mm\leq W\leq 7\;mm\\ 1mm\leq B\leq 7mm\\ 1mm\leq H\leq 5mm \end{array}\right.$$

In the equations, *Y_1_*, *Y_2_*, and *Y_3_* represent the response surface model expressions. These expressions describe the first-order natural frequency, the maximum deformation at the housing base, and the mass of the beam combining module as functions of the design variables *W*, *B*, and *H*. The lower limit for the first-order natural frequency and the upper limit for the maximum deformation at the housing base are derived from the modal and random vibration analysis data of the original structure, ensuring a benchmark for optimization. The upper limit for the housing mass is set after fully considering the weight sensitivity of the final application platform. The value ranges of the design variables are primarily determined based on the geometric feasibility of the housing base, manufacturing process, and the mass upper limit of the beam combining module.

**Figure 7:**
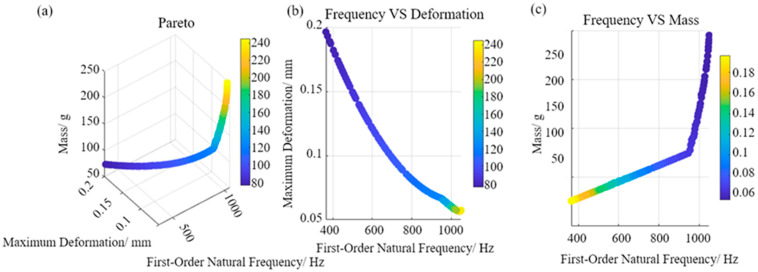
Pareto optimization results. (**a**) Distribution of the Pareto optimal front after 500 iterations; (**b**) relationship between frequency and deformation; (**c**) relationship between frequency and mass.

The key parameters of the multi-objective PSO algorithm are set with specific values. The population size is 100. The number of generations is 500. The inertia weight is initialized to 0.7. It is adaptively adjusted using a decay factor of 0.99. Both the individual and social learning factors are set to 1.5. This parameter combination is selected to effectively balance the algorithm’s global exploration and local exploitation capabilities. The inertia weight controls the inheritance of a particle’s current velocity. Its initial value of 0.7 maintains strong global search capability. This helps avoid premature convergence. A decay factor of 0.99 gradually reduces the weight over iterations. This reduction enhances local fine search in later stages. Consequently, it improves convergence precision. Setting the learning factors to 1.5 ensures equal consideration of personal best and global best. This promotes balance between population diversity and convergence. The described parameter configuration enables stable and efficient solution space search. It yields a well-distributed Pareto front, as shown in Figure 7a. Excessively high inertia weights or overly small learning factors may prevent convergence to high-quality solutions. Conversely, opposite conditions can lead to convergence to local optima.

Figure 7a illustrates the distribution of optimal solutions within a three-dimensional objective space. This space is defined by the first-order natural frequency, maximum deformation, and mass. The solution set forms a smooth and continuous non-dominated surface. This indicates full algorithm convergence after 500 iterations. The uniform distribution of solution points along the surface demonstrates the effectiveness of the crowding distance mechanism. This mechanism successfully maintains diversity and spread within the solution set. Figure 7b depicts the relationship between the module’s first-order natural frequency and deformation during optimization. It reveals a strong negative correlation. An increase in the first-order natural frequency leads to a significant reduction in maximum deformation under random vibration. The stiffness enhancement directly reduces the module’s dynamic response under identical excitation. This clearly manifests the vibration resistance improvement achieved through structural optimization. Figure 7c shows the relationship between the module’s first-order natural frequency and its mass. It exhibits a distinct positive correlation. This indicates that pursuing higher natural frequency typically increases structural mass. This relationship highlights a common conflict in engineering optimization between performance and lightweight design. It quantifies the mass penalty associated with improved vibration resistance. This quantification is crucial for making informed design decisions under specific weight constraints.

The Pareto multi-objective optimization based on the CCD-PSO approach has been completed. Three representative extreme candidate solutions were extracted from the Pareto front. Their corresponding stiffener structural parameters and key performance metrics are listed in Table 11. Solution 1 is located in the region of optimal compromise on the trade-off surface. It significantly suppresses vibrational deformation while achieving the best performance for *Y_2_*. This solution raises the first-order natural frequency above 1036.1 Hz with a controlled mass increase. These results demonstrate the effectiveness of multi-objective collaborative optimization. Solution 2 represents a design philosophy that prioritizes stiffness enhancement absolutely. It achieves a frequency close to 1000 Hz but at the cost of increased mass. The mass increases to approximately 250 g. This highlights the acute conflict between performance and lightweight design. Solution 3 exemplifies an extreme lightweight design. Its mass is only about 75 g. However, its first-order natural frequency remains around 365 Hz. This frequency is still below the 400 Hz safety threshold. This proves that weight reduction alone cannot meet vibrational reliability requirements. In summary, Solution 1 is selected as the final implementation scheme. It achieves the best compromise between vibration suppression and mass control while satisfying the frequency constraint. The corresponding optimal parameter combination is *W* = 4.8 mm, *B* = 4.6 mm, *H* = 5 mm.

### 5.5. Simulation Verification and Performance Evaluation

**Figure 8:**
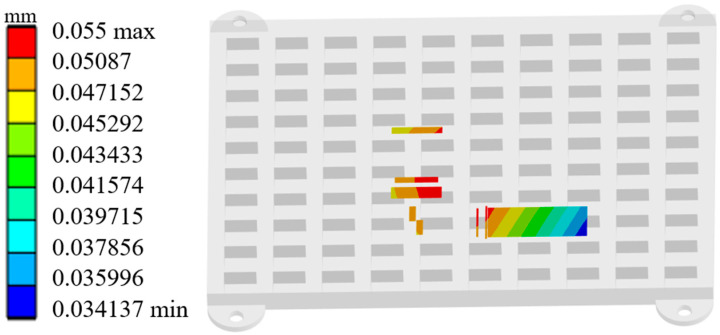
Deformation at critical locations of the beam combining module under vibration testing with optimal parameters.

Since the optimization results from the algorithm are predicted values, the optimal solution’s effectiveness required validation. This validation was conducted through simulation. In the simulation test, the first-order natural frequency of the beam combining module was measured at 1036.1 Hz. The relative error for this measurement was only 0.12%. Figure 8 shows the deformation schematic of key locations on the optimally designed module housing under vibration testing. The maximum deformation recorded was 0.055 mm. This deformation value corresponds to a relative error of 3.09%. The mass was measured at 214.97 g, with a relative error of 0.14%. These results confirm the reliability of the derived Pareto optimal solution. Compared to the pre-optimization design, the module’s first-order natural frequency increased by 679.8 Hz. Simultaneously, the deformation at the critical location under vibration testing decreased by 0.2068 mm. The mass increase was limited to only 165.47 g. This optimization outcome verifies the effectiveness and reliability of the CCD-PSO method for housing vibration resistance optimization problems. Achieving equivalent vibration resistance solely by increasing the housing base thickness would have different consequences. Such an approach would result in a mass increase exceeding 270 g. This significant weight gain would substantially limit the widespread application of semiconductor laser beam combining modules.

**Figure 9:**
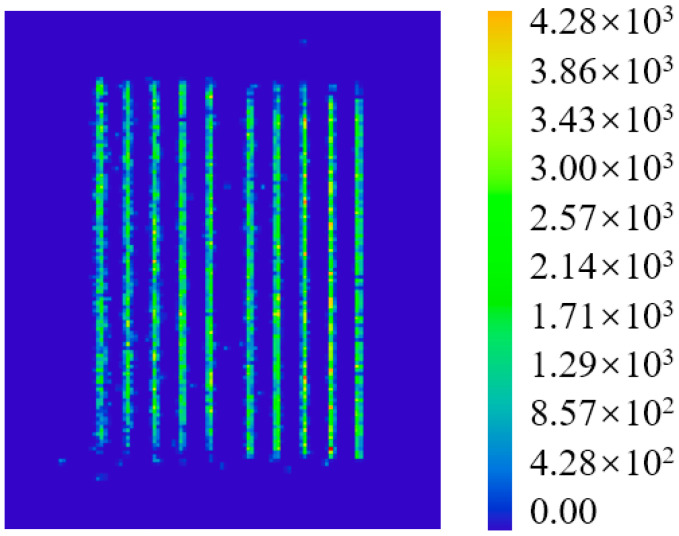
Non-coherent irradiance of the output laser from the optimized beam combining module.

To verify the reliability of the optimized beam combining module under vibration conditions through simulation, this study established a random vibration analysis procedure in finite element software according to the GJB 150.16A-2009 standard. The dynamic displacement response at critical locations of the beam combining module (specifically the fast-axis collimating lens) was extracted through simulation, and this displacement data was imported into optical design software for optical path coupling analysis, thereby calculating the changes in laser power and irradiance. The results indicated that the combined power decreased from 300 W before testing to 290 W, representing a reduction of only 3.3%. Furthermore, the peak irradiance of the output laser from the beam combining module decreased from 4520 W/cm^2^ to 4280 W/cm^2^, a modest decline of 5.3%. Figure 9 shows the incoherent irradiance of the output laser from the optimized beam combining module These findings demonstrate that the optimization scheme effectively ensures the reliability of the semiconductor laser under vibrational environments.

A portable closed-loop design methodology based on CCD and PSO is established. The core workflow follows three main steps. First, CCD collects a finite set of experimental sample data. This data constructs a high-precision quadratic response surface surrogate model. The model efficiently maps relationships between housing structural parameters and key performance indicators. The maximum error between predicted and actual values is strictly controlled within 1.142%. Next, the Multi-Objective PSO algorithm performs optimization. It conducts a global Pareto front search within the surrogate model’s continuous solution space. This process effectively balances three objectives: frequency enhancement, displacement control, and mass constraint. Finally, high-confidence solutions undergo simulation validation. This allows for iterative model refinement. The method successfully circumvents subjective bias in traditional trial-and-error approaches. It reduces experimental iteration costs by over 40%. The approach maintains high-fidelity accuracy with errors controlled within 3.09%. The “modeling-optimization-validation” framework demonstrates high transferability. It can be rapidly deployed for vibration-sensitive opto-electronic systems through parameter adjustments. Applications include semiconductor laser diode array beam combining systems. Other applications are high-precision optical shaping modules and integrated opto-mechanical devices. The methodology efficiently generates Pareto optimal solution sets. It significantly reduces experimentation and prototyping costs. It also shortens development cycles. This provides a scalable optimization paradigm with broad engineering applicability. The paradigm serves advanced optoelectronic device packaging and vibration-resistant design of high-precision opto-mechanical systems.

The combined CCD-PSO method has achieved favorable results for regular housing structures. It offers an efficient strategy for vibration-resistant design of similar opto-mechanical systems. However, its application boundaries and future extensions warrant further discussion. A primary limitation involves the CCD response surface model. This model demonstrates high accuracy within the validated design space. However, extrapolation requires caution. Future work could integrate adaptive sampling techniques. Advanced surrogate modeling methods could also improve extrapolation capability and generalizability. Regarding the PSO algorithm, the parameter settings proved effective in this study. However, algorithm performance remains problem-dependent. Subsequent research should investigate this through sensitivity analysis. Adaptive parameter mechanisms could enhance robustness. This study validated the method using fundamental geometric configurations. This foundation supports handling more complex structures. Future work could integrate this framework with parametric modeling. Multiphysics coupling analysis could extend applications to multidisciplinary design optimization. Such integration would significantly increase applicability and engineering value for high-end optoelectronic devices.

## 6. Conclusions

This study aims to enhance the vibration reliability of semiconductor laser diode array beam combining systems under service conditions. A structural-vibration coupling model is established to numerically evaluate the impact of housing stiffener structures on the module’s dynamic characteristics. Furthermore, a portable closed-loop design methodology based on CCD and PSO is proposed. This methodology employs RSM to establish mathematical relationships. These relationships connect stiffener structural parameters to key module performance indicators: the first-order natural frequency, maximum vibration deformation, and mass. The multi-objective particle swarm algorithm is then applied to optimize the stiffener parameters. This optimization determines the optimal combination of key structural parameters for the housing stiffeners. The main conclusions are as follows:

(1) The failure characteristics of semiconductor laser diode array beam combining systems under vibrational environments were investigated. The first-order natural frequency of the beam combining module is 356.3 Hz, making it prone to resonance under complex vibrational excitation. This resonance induces a deformation of 0.2618 mm at critical mounting locations, significantly exceeding the 0.16 mm focal length tolerance of the fast-axis collimating lens. Consequently, the optical path is severely misaligned, causing the combined power to plummet from 300 W to 1.5 W. These results confirm that excessive deformation due to insufficient structural stiffness is the core failure mechanism for such systems in vibrational environments.
(2) A reinforcement design based on response surface methodology and multi-objective optimization. CCD was used to establish response surface models relating the stiffener width *W*, width *B*, and thickness *H* to the beam combining module’s first-order natural frequency, maximum deformation, and mass. The model’s coefficient of determination (*R^2^*) reached 0.9996, indicating an excellent fit. Multi-objective PSO was applied to determine the optimal combination of key stiffener parameters: *W* = 4.8 mm, *B* = 4.6 mm, and *H* = 5 mm. The error between the method’s predicted values and the simulation results remained below 3.1%, validating the model’s effectiveness.
(3) Structural optimization has achieved a leapfrog improvement in the vibration reliability of the semiconductor laser diode array beam combining module. For the optimal parameter combination, the first-order natural frequency was significantly increased to 1036.1 Hz (a 290.7% improvement over the original design), and the maximum deformation at critical locations was suppressed to 0.055 mm (a 79.0% reduction from the original value). Vibration testing verified the optimization effectiveness, showing only a 3.3% decrease in output laser power and stable incoherent irradiance. Meanwhile, the mass increase was controlled within a reasonable range, achieving a balance between vibration resistance and lightweight design, which demonstrates the efficacy of the proposed solution.

This study investigates the dynamic response of the semiconductor laser diode array beam combining module under vibrational environments and proposes an optimization scheme to enhance its vibration reliability. The results demonstrate that the combined CCD-PSO method exhibits significant advantages in housing anti-vibration design. It achieves remarkable improvement in vibration resistance through high-precision response surface models and intelligent search algorithms, while substantially reducing experimental costs and development cycles, demonstrating strong engineering applicability and transferability. However, limitations remain in the reliability of model extrapolation predictions and the adaptability of algorithm parameters to complex problems, particularly the generalizability for irregular structures or multi-physics coupling scenarios. Future research could focus on integrating adaptive sampling techniques to enhance the generalization of surrogate models, and combining parametric modeling with topology optimization and thermo-mechanical-optical multi-physics coupling analysis frameworks to further expand the universality and practical value of this method in complex systems such as high-end optoelectronic devices.

## Figures and Tables

**Table 1:** Semiconductor laser diode array beam combining module housing material parameters.

Material	Density (Kg/m^3^)	Young’s Modulus (10^9^ Pa)	Poisson Ratio	Shear Modulus (10^9^ Pa)	Bulk Modulus (10^9^ Pa)
6061-T6	2713	69.04	0.33	25.955	67.686

**Table 2:** Simulated package properties vs. mesh count.

Mesh Count	First-Order Natural Frequency (Hz)	Maximum Deformation (mm)	First-Order Natural Frequency Error (%)	Maximum Deformation Error (%)
26,926	359.6	0.2585	1.0396	0.1899
46,078	358.2	0.25978	0.6462	0.6860
62,671	357.4	0.26053	0.4214	0.9766
101,490	356.3	0.2571	0.1123	0.3527
183,024	355.9	0.25801	Benchmark	Benchmark

**Table 3:** First six natural frequencies of the semiconductor laser diode array beam combining module.

Order	1st	2nd	3rd	4th	5th	6th
Frequency/**Hz**	356.3	731.05	847.45	1020.5	1620.9	1793

**Table 4:** Acceleration power spectral density.

Frequency/Hz	PSD
20	0.040 g^2^/Hz
1000–2000	−6 dB/octave
2000	0.010 g^2^/Hz

**Table 5:** Design variable parameters.

Factor	−α	−1	0	1	α
*W*/mm	2.2	3	5	7	7.8
*B*/mm	2.2	3	5	7	7.8
*H*/mm	1.6	2	3	4	4.4

**Table 6:** Design and results of the CCD scheme.

Run	*W*/mm	*B*/mm	*H*/mm	*Y*_1_/Hz	*Y*_2_/mm	*Y*_3_/g
1	5	5	3	761.26	0.084389	164.06
2	5	5	3	761.23	0.084391	164.06
3	5	5	3	761.28	0.084387	164.06
4	7	7	4	914.44	0.063043	218.57
5	7	7	2	634.56	0.11027	149.04
6	7	3	4	891.35	0.066578	207.36
7	7	3	2	616.36	0.1164	143.44
8	3	7	4	879.77	0.067351	187.86
9	3	7	2	600.36	0.1205	133.68
10	3	3	4	869.23	0.069752	155.2
11	3	3	2	578.08	0.12895	117.35
12	7.8	5	3	768.8	0.082636	185.82
13	2.2	5	3	714.77	0.092932	142.31
14	5	7.8	3	768.89	0.082444	175.58
15	5	2.2	3	737.4	0.089758	152.55
16	5	5	4.4	957.78	0.059272	203.52
17	5	5	1.6	558.55	0.13471	124.6

**Table 7:** ANOVA for first-order natural frequency model.

Source	Sum of Squares	df	Mean Square	F-Value	*p*-Value
Model	2.434 × 10^05^	9	27,044.67	2107.51	<0.0001
*W*	3522.56	1	3522.56	274.50	<0.0001
*B*	1172.00	1	1172.00	91.33	<0.0001
*H*	2.380 × 10^05^	1	2.380 × 10^05^	18,547.20	<0.0001
*WB*	8.97	1	8.97	0.6988	0.4308
*WH*	30.77	1	30.77	2.40	0.1654
*BH*	5.87	1	5.87	0.4571	0.5207
*W^2^*	597.80	1	597.80	46.59	0.0002
*B^2^*	77.49	1	77.49	6.04	0.0436
*H^2^*	3.57	1	3.57	0.2778	0.6144
Residual	89.83	7	12.83		
Lack of Fit	89.83	5	17.97		
Pure Error	0.0006	2	0.0003		
Cor Total	2.435 × 10^05^	16			

**Table 8:** ANOVA for maximum deformation model of housing base.

Source	Sum of Squares	df	Mean Square	F-Value	*p*-Value
Model	0.0089	9	0.0010	1919.57	<0.0001
*W*	0.0002	1	0.0002	324.90	<0.0001
*B*	0.0001	1	0.0001	153.97	<0.0001
*H*	0.0083	1	0.0083	16152.44	<0.0001
*WB*	1.758 × 10^−07^	1	1.758 × 10^−07^	0.3412	0.5775
*WH*	2.925 × 10^−05^	1	2.925 × 10^−05^	56.76	0.0001
*BH*	9.340 × 10^−06^	1	9.340 × 10^−06^	18.12	0.0038
*W^2^*	1.698 × 10^−05^	1	1.698 × 10^−05^	32.95	0.0007
*B^2^*	3.250 × 10^−06^	1	3.250 × 10^−06^	6.31	0.0403
*H^2^*	0.0003	1	0.0003	547.80	<0.0001
Residual	3.608 × 10^−06^	7	5.154 × 10^−07^		
Lack of Fit	3.608 × 10^−06^	5	7.215 × 10^−07^		
Pure Error	8.000 × 10^−12^	2	4.000 × 10^−12^		
Cor Total	0.0089	16			

**Table 9:** ANOVA for mass model of housing base.

Source	Sum of Squares	df	Mean Square	F-Value	*p*-Value
Model	13,558.29	6	2259.72	1573.04	<0.0001
*W*	2878.49	1	2878.49	2003.78	<0.0001
*B*	806.40	1	806.40	561.35	<0.0001
*H*	9469.34	1	9469.34	6591.81	<0.0001
*WB*	129.44	1	129.44	90.11	<0.0001
*WH*	214.45	1	214.45	149.28	<0.0001
*BH*	60.17	1	60.17	41.89	<0.0001
Residual	14.37	10	1.44		
Lack of Fit	14.36	8	1.80		
Pure Error	0.0005	2	0.0002		
Cor Total	13,572.66	16			

**Table 10:** Correlation coefficients of the target model.

Target Model	First-Order Natural Frequency	Maximum Deformation	Mass
*R^2^*	0.9996	0.9996	0.9989
*R^2^* (Predicted)	0.9992	0.9991	0.9983
*R^2^* (Adjusted)	0.9964	0.9960	0.9893

**Table 11:** Pareto optimization design results.

Scheme	*W*/mm	*B*/mm	*H*/mm	First-Order Natural Frequency/Hz	Maximum Deformation/mm	Mass/g
1	4.8	4.6	5	1037.359	0.0567	215.27
2	6.04	6.90	5	1050.18	0.0573	244.94
3	1	1.04	1	365.12	0.1961	75.99

## Data Availability

The original contributions presented in this study are included in the article; further inquiries can be directed to the corresponding authors.

## Abbreviations

The following abbreviations are used in this manuscript:

CCD	Central Composite Design
PSO	Particle Swarm Optimization
PSD	Power Spectral Density
ANOVA	Analysis of Variance
RSM	response surface methodology
